# Multicenter research priorities in pediatric CMR: results of a collaborative wiki survey

**DOI:** 10.1038/s41598-023-34720-9

**Published:** 2023-06-03

**Authors:** Rebecca S. Beroukhim, Anthony Merlocco, Jennifer F. Gerardin, Edythe Tham, Jyoti K. Patel, Saira Siddiqui, Benjamin Goot, Kanwal Farooqi, Jonathan Soslow, Heynric Grotenhuis, Kan Hor, Vivek Muthurangu, Francesca Raimondi

**Affiliations:** 1 Department of Cardiology, Harvard Medical School, Boston Children’s Hospital, 300 Longwood Avenue, Boston, MA 02115 USA; 2 Department of Cardiology, University of Tennessee Health Science Center, Le Bonheur Children’s Hospital, Memphis, TN USA; 3 Division of Pediatric Cardiology, Medical College of Wisconsin, Children’s Hospital of Wisconsin, Milwaukee, WI USA; 4 Department of Pediatrics, Stollery Children’s Hospital, University of Alberta, Edmonton, AB Canada; 5 Division of Cardiology, Department of Pediatrics, Riley Hospital for Children at Indiana University Health, Indianapolis, IN USA; 6 Division of Pediatric Cardiology, Atlantic Health System, Morristown, NJ USA; 7 Division of Pediatric Cardiology, Department of Pediatrics, Columbia University Medical Center, New York Presbyterian-Morgan Stanley Children’s Hospital, New York, NY USA; 8 Division of Pediatric Cardiology, Department of Pediatrics, Vanderbilt University, Nashville, TN USA; 9 Department of Pediatric Cardiology, Utrecht Medical Center, Utrecht, The Netherlands; 10 Department of Pediatrics, The Heart Center, Nationwide Children’s Hospital, Ohio State University, Columbus, OH USA; 11 Department of Cardiology, UCL Center for Translational Cardiovascular Imaging, University College London, London, UK; 12 Department of Cardiology, Meyer Children’s Hospital, University of Florence, Florence, Italy

**Keywords:** Cardiology, Magnetic resonance imaging, Paediatric research

## Abstract

Multicenter studies in pediatric cardiovascular magnetic resonance (CMR) improve statistical power and generalizability. However, a structured process for identifying important research topics has not been developed. We aimed to (1) develop a list of high priority knowledge gaps, and (2) pilot the use of a wiki survey to collect a large group of responses. Knowledge gaps were defined as areas that have been either unexplored or under-explored in the research literature. High priority goals were: (1) feasible and answerable from a multicenter research study, and (2) had potential for high impact on the field of pediatric CMR. Seed ideas were contributed by a working group and imported into a pairwise wiki survey format which allows for new ideas to be uploaded and voted upon (https://allourideas.org). Knowledge gaps were classified into 2 categories: ‘Clinical CMR Practice’ (16 ideas) and ‘Disease Specific Research’ (22 ideas). Over a 2-month period, 3,658 votes were cast by 96 users, and 2 new ideas were introduced. The 3 highest scoring sub-topics were myocardial disorders (9 ideas), translating new technology & techniques into clinical practice (7 ideas), and normal reference values (5 ideas). The highest priority gaps reflected strengths of CMR (e.g., myocardial tissue characterization; implementation of technologic advances into clinical practice), and deficiencies in pediatrics (e.g., data on normal reference values). The wiki survey format was effective and easy to implement, and could be used for future surveys.

## Introduction

Pediatric cardiovascular magnetic resonance (CMR) has unique strengths in the evaluation of children with congenital and acquired heart disease, allowing for comprehensive evaluation of anatomy, physiology, and tissue characterization^1^. Standard examinations that include anatomical surveys, quantification of ventricular size and function, and late gadolinium enhancement imaging are routinely used in clinical practice to guide medical and surgical management of children with heart disease. In addition, novel imaging sequences such as parametric mapping techniques are frequently studied to determine applicability in pediatrics. Fibrosis, the final common pathway of myocardial diseases from a variety of insults, is associated with abnormal myocardial remodeling, worsening ventricular function, and ultimately increased mortality^2–5^. Although myocardial biopsy has traditionally been the gold standard for detection of fibrosis, it is an invasive procedure associated with morbidity and mortality, prone to sampling error, and does not evaluate the entire myocardium^2,6^. Parametric tissue mapping by CMR has the potential to substitute as a ‘noninvasive myocardial biopsy’, which may benefit patients with these serious and sometimes fatal myocardial disorders^7,8^.

**Figure 1:**
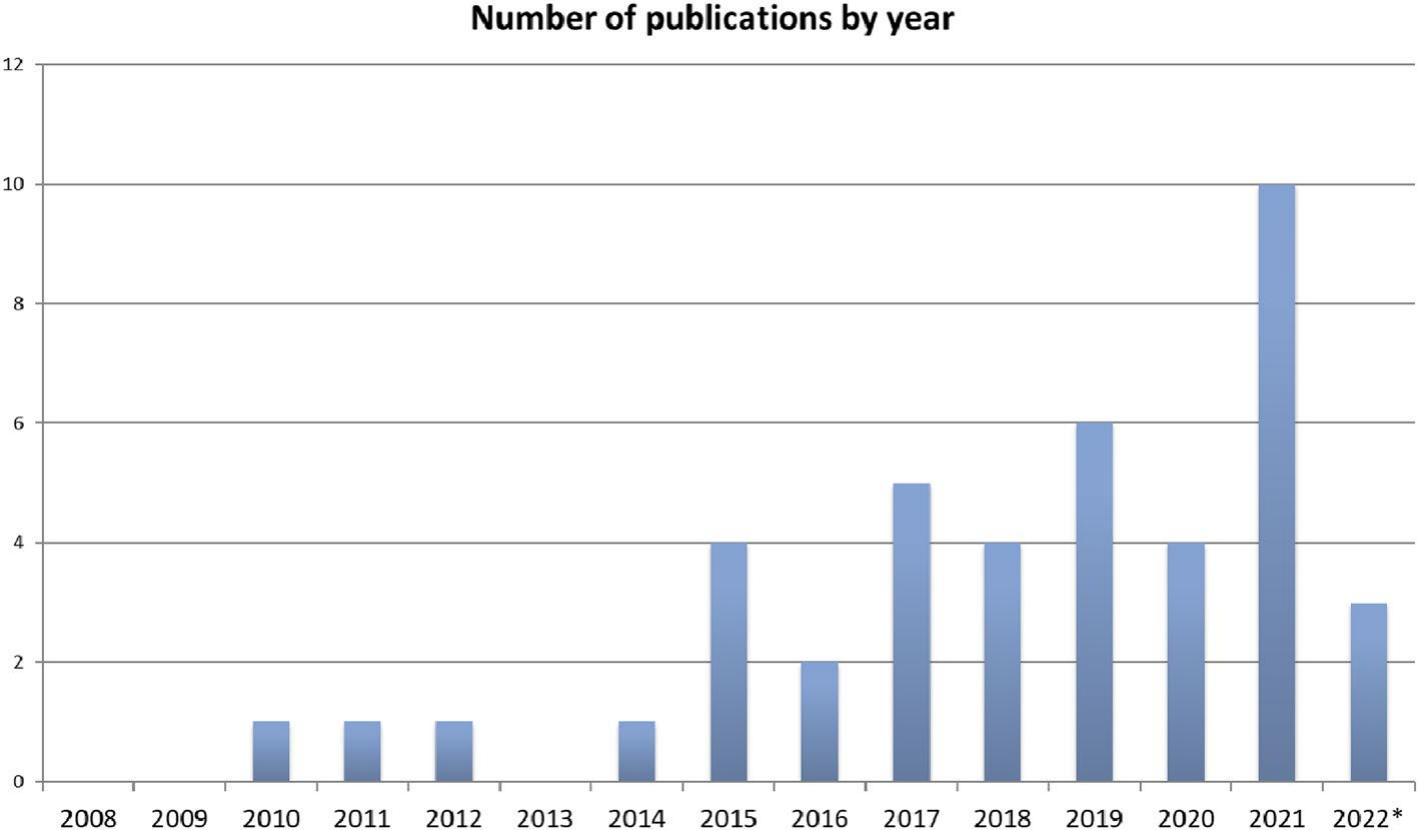
Number of multicenter pediatric CMR publications by year. Data was extracted from a Pubmed search for CMR studies in children, and multicenter was defined as ≥ 2 centers. *Jan-April 2022.

However, many pediatric cardiac diseases are rare enough that meaningful data cannot be obtained from single institution studies. Furthermore, the requirement for sedation in young children (approximately 0–6 years of age) limits the ability to capture normal reference values^9,10^. Consequently, studies aimed at supporting patient management strategies and outcome prediction are often underpowered. Therefore, multicenter studies are urgently needed to inform management and improve outcomes in this population. While the volume of multicenter pediatric CMR publications appears to have increased over time, the total number of publications remains low (Fig. 1). Although multicenter research is by definition collaborative, study aims are historically selected based on the interests of individual investigators, rather than by group consensus. In this project, we aimed to (1) develop a list of high priority knowledge gaps in pediatric multicenter CMR research that may serve as a guide to future investigators, and (2) develop a structured, efficient, and fair process by which important knowledge gaps can be identified in the future. We chose a wiki survey format (All Our Ideas; https://allourideas.org), which is an intentional open, crowd-source design that allows for a range in number of responses, and allows for new knowledge gaps to be contributed to the survey by respondents. We hope that this effort will serve as a springboard to advance the field of pediatric CMR.

## Methods

**Figure 2:**
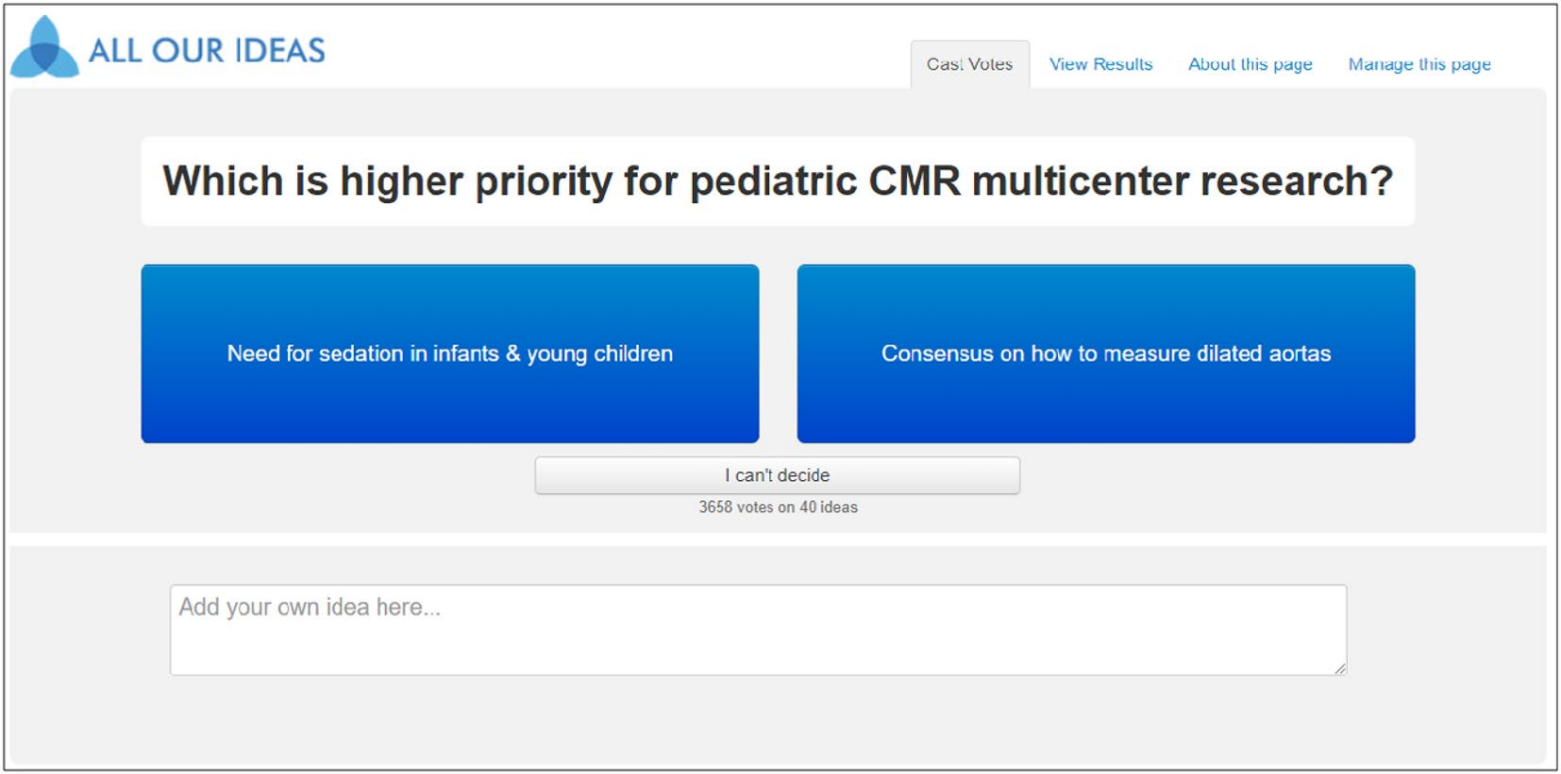
Format of the pairwise wiki survey. After logging into the survey, participants are asked to vote for the higher priority idea, or to upload a new idea. After voting, the page refreshes and presents another pairwise comparison. Participants can vote as many or as little times as they wish, and are given the option of viewing survey results in real time. After logging out, they can log back in for a new user session. All responses are anonymous.

A working group of 13 pediatric CMR investigators involved in the Society for Cardiovascular Magnetic Resonance (SCMR) convened to develop seed ideas for multicenter research that would fill important knowledge gaps in the literature. Group members had been actively involved in the Multicenter Collaborative Research Subcommittee of the Pediatrics and Congenital Heart Disease Special Interest Group, and this initiative was intended to spark interest and help guide multicenter research. There was nearly equal gender representation (6 male, 7 female), with relatively wide practice representation including cardiologists from small academic centers, large academic centers, and private practice. Knowledge gaps were defined as research priorities that have not been explored or have been under-explored. Topics such as practice variation among institutions or quality improvement activities were excluded because the topics were too closely associated with research ideas and difficult to compare with each other (e.g. Research idea: “TOF ACHD consensus guidelines on PVR: applicable in children and adolescents” vs. Practice variation/quality improvement idea: “Use of TOF ACHD consensus guidelines on PVR”. Research goals were considered high priority if they were (1) feasible and answerable from a multicenter study, and (2) of high impact in the field of pediatric CMR. Ideas were organized, edited, and discussed over the course of several planned zoom meetings. Ideas were also actively solicited from other members of the Pediatrics and Congenital Heart Disease Special Interest Group during online education webinars and meetings (Table 1). Based on consensus, 38 seed ideas were uploaded into a unique “wiki survey” format (‘All Our Ideas’; https://allourideas.org) for distribution to a larger group of pediatric CMR stakeholders. A pilot wiki survey was administered to the working group to predict the number and range of responses to the survey, and to compare with the final survey responses. The final survey was advertised through multiple sources, including email distribution lists, social media, and at the Society for Cardiovascular Magnetic Resonance (SCMR) annual meeting in February 2022. Respondents contributed anonymously to the wiki survey, which asks for pairwise comparisons between items (Fig. 2), and allows for addition of new ideas that can be presented to future respondents. Respondents were allowed to respond “I can’t decide” if they felt that both research ideas were of equal priority. Participants could log into multiple sessions to continue voting or uploading new ideas, from January 24, 2022 through March 3, 2022. This study was approved by the Institutional Review Board at Boston Children’s Hospital, and the survey was performed in accordance with relevant guidelines and regulations. Informed consent was obtained from all participants.

**Table 1:** Classification of 40 ideas (# ideas per category) contributed to the wiki survey.

**Clinical CMR Practice**
Normal reference values (5)
Defining cutoffs for mild, moderate, severe (3)
Reproducibility (3)
Translating new technologies & techniques into clinical practice (7)
**Disease Specific Research**
Myocardial disorders (e.g., cardiomyopathy, myocarditis, transplant) (9)
Rare diseases (e.g., rare congenital lesions, cardiac masses) (1)
Valvular disorders (e.g., Ebstein anomaly, bicuspid aortic valve) (0)
Arterial lesions (e.g., coarctation of the aorta, coronary artery anomalies, aortopathy) (2)
Conotruncal lesions (e.g., tetralogy of Fallot, truncus arteriosus) (3)
Complex congenital heart disease (e.g., single ventricle or borderline ventricle heart disease) (4)
Shunting lesions (e.g., atrial and ventricular septal defects) (0)
Pulmonary hypertension (3)

‘Clinical CMR Practice’ = research that guides the collection and interpretation of data, and ‘Disease Specific Research’ = research specific to congenital or acquired diagnoses.

## Results

Knowledge gaps identified by the working group were classified into 2 broad categories: ‘Clinical CMR Practice’ (16 seed ideas) and ‘Disease Specific Research’ (22 seed ideas) (Table 1). ‘Clinical CMR Practice’ refers to research that guides the collection and interpretation of data, and ‘Disease Specific Research’ includes research specific to congenital or acquired diagnoses. From the pilot survey, we cast 535 votes over 5 min during a planning meeting; this resulted in approximately 8 votes per minute per person. The categories that received the highest scores included myocardial disorders, complex congenital heart disease (CHD), normal reference values, conotruncal lesions, and translating technologies & techniques into clinical practice (Supplemental Table 1, Supplemental Figure 1). From this pilot data, we predicted that 100 members would vote for 4 min each, with approximately 3,200 votes in the final survey.

**Figure 3:**
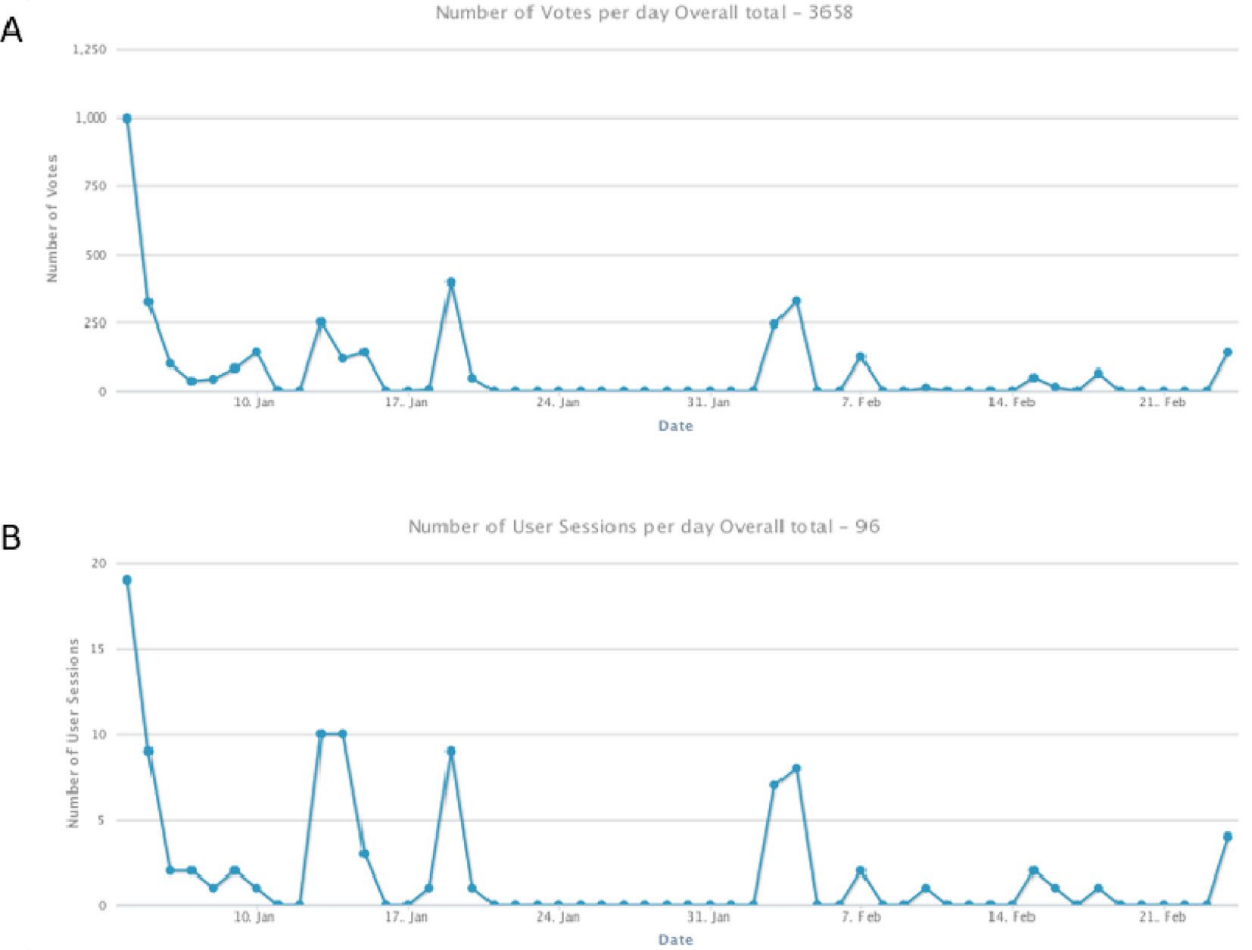
Number of votes and user sessions per day, during the voting period from January 24, 2022 through March 3, 2022.

**Figure 4:**
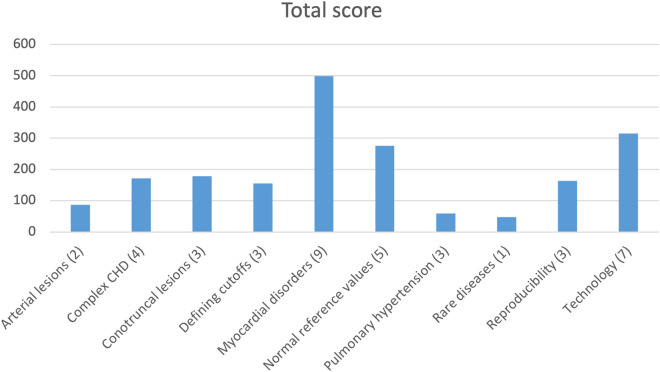
Combined scores based on classification. The x-axis denotes topic (#ideas), and the bar represents the combined score for all topics in that category.

The final survey was activated over a 2-month period, with 3,658 votes cast among 96 user sessions for an average of 38 votes per user session (Fig. 3). Events that correlated with higher voting included specific requests to SCMR pediatric/congenital section members: January 4 (email request); January 13 (steering committee meeting); January 19 (webinar); February 3–4 (annual scientific sessions). Of 40 ideas that received votes, 38 were seed ideas contributed by the working group, and 2 were uploaded by participants (‘Artificial intelligence and machine learning – developing a backbone to improve post-processing’, and ‘Artificial intelligence and machine learning – developing a backbone to improve prognostication’). Two additional ideas contributed by participants were not activated because they were duplicates of seed ideas. Scores for individual ideas are shown in Table 2, and the combined scores for each classification are displayed in Fig. 4. The three major topics that received the highest scores included myocardial disorders, technology, and normal reference values.

**Table 2:** Multicenter research priorities with rankings and classification of research area.

Multicenter research priority		Score	Classification
1	Normal parametric imaging values in children by CMR and multivendor reproducibility	75	Normal reference values
2	TOF ACHD consensus guidelines on PVR: applicable in children and adolescents?	70	Conotruncal lesions
3	Noninvasive detection of cardiac transplant rejection in children	70	Myocardial disorders
4	CMR criteria for biventricular repair in borderline left ventricles	68	Complex CHD
5	Normal ventricular mass and volumes in children	67	Normal reference values
6	Outcome after detection of LGE in children with myocarditis	65	Myocardial disorders
7	TOF ACHD consensus guidelines on use of CMR: applicable in children and adolescents?	64	Conotruncal lesions
8	Normal vascular dimensions in children	62	Normal reference values
9	Prognostic value of myocardial tissue mapping in myocarditis	60	Myocardial disorders
10	Reproducibility of ventricular measurements (function, mass) across institutions	61	Reproducibility
11	Dilated cardiomyopathy: CMR based predictors of outcome	58	Myocardial disorders
12	CMR predictors of outcome following the Fontan operation	58	Complex CHD
13	Prognostic value of CMR in pediatric cancer survivors	57	Myocardial disorders
14	Need for sedation in infants and young children	57	Implementing new technologies & techniques into clinical practice
15	Clinical utility of 4D flow	56	Implementing new technologies & techniques into clinical practice
16	Safety of CMR imaging of children with pacemakers and defibrillators	54	Implementing new technologies & techniques into clinical practice
17	Consensus on how to measure dilated aortas	53	Defining cutoffs for mild, moderate, severe
18	Reproducibility of LGE quantification across institutions	52	Reproducibility
19	Prognostic value of myocardial tissue mapping in HCM	52	Myocardial disorders
20	Standardized cutoffs for aortic regurgitation and association with outcomes	51	Defining cutoffs for mild, moderate, severe
21	Development of a pediatric risk calculator in HCM	51	Myocardial disorders
22	Standardized cutoffs for AVV regurgitation and association with outcomes	51	Defining cutoffs for mild, moderate, severe
23	Stress CMR: institutional variation in stress, imaging protocols, interpretation	50	Reproducibility
24	Stress CMR and LGE for assessment of anomalous aortic origin of a coronary artery (AAOCA)	49	Arterial lesions
25	Parametric tissue mapping in the diagnosis of cardiac tumors and masses	48	Rare diseases
26	Does veno-venous collateral burden predict desaturation and exercise capacity in Fontan patients?	46	Complex CHD
27	Prognostic value of CMR in Duchenne muscular dystrophy-associated cardiomyopathy	45	Myocardial disorders
28	Stress CMR after arterial switch operation in D-TGA	44	Conotruncal lesions
29	Sensitivity and specificity of CMR for detection of ARVC in children	41	Myocardial disorders
30	CMR predictors of adverse outcome after the double switch operation in L-TGA	40	Complex CHD
31	Normal atrial size in children	40	Normal reference values
32	Lymphatic MR imaging: developing pathways for local implementation	40	Implementing new technologies & techniques into clinical practice
33	Artificial intelligence and machine learning – developing a backbone to improve post-processing*	40	Implementing new technologies & techniques into clinical practice
34	CMR predictors of adverse ventricular remodeling in coarctation of the aorta	38	Arterial lesions
35	Artificial intelligence and machine learning – developing a backbone to improve prognostication*	36	Implementing new technologies & techniques into clinical practice
36	Clinical utility of fetal CMR	32	Implementing new technologies & techniques into clinical practice
37	Nomogram for dilated aorta in Turner syndrome	31	Normal reference values
38	CMR-guided catheterization for assessment of pulmonary arterial hypertension	29	Pulmonary hypertension
39	Is exercise CMR prognostic in children with pulmonary arterial hypertension?	22	Pulmonary hypertension
40	CMR before and after the Potts shunt for pulmonary arterial hypertension	8	Pulmonary hypertension

*Ideas uploaded by survey participants. *CMR* Cardiovascular magnetic resonance, *TOF* Tetralogy of Fallot, *ACHD* Adult congenital heart disease, *HCM* Hypertrophic cardiomyopathy, *AVV* Atrioventricular valve, *LGE* Late gadolinium enhancement, *AAOCA* Anomalous aortic origin of a coronary artery, *D-TGA* D-transposition of the great arteries, *ARVC* Arrhythmogenic right ventricular cardiomyopathy, *L-TGA* L-transposition of the great arteries, *CHD* Congenital heart disease.

## Discussion

Multicenter research is integral to addressing major knowledge gaps in pediatric CMR. By combining data across institutions, studies have both improved statistical power and generalizability. However, such efforts require additional resources, collaboration among investigators, and strict legal regulation between institutions. Therefore, appropriate selection of research ideas is paramount in order to meet the needs of the group at large. We embarked on the pediatric CMR gap analysis to identify the most important multicenter research needs in the current era, and to develop a structured process for future collection of potential research ideas.

We piloted a “wiki survey” format, which allows respondents to vote anonymously on seed ideas, upload new ideas, and view results in real time. The wiki survey was chosen due to a number of strengths that are not available in other survey formats. Traditional survey methods (e.g. questionnaires, interviews, and panel surveys) have a set of closed questions that may lead to missed opportunities because respondents ‘true’ answers may fall outside of the researcher-created answer choices, or the respondents may decide that the survey is too lengthy and opt out. The Delphi method (a systematic method that relies on a panel of experts) was not considered because of the closed process and bureaucratic nature. Rather, we aimed for a democratic, structured analysis of priorities amongst the larger group, which provided an opportunity to promote idea-generation and involve all members of the subcommittee. All Our Ideas wiki survey is an intentional open, crowd-source design that allows for the survey to be greedy (collects as much or as little information as each participant is willing to provide), and collaborative (allows open or new information contributed directly by respondents. Furthermore, statistical methods are applied to mitigate the effects of unequal responses between users, as there are typically a few ‘heavy’ users and a larger proportion of ‘light’ users. Based on responses given, the software analyzes the data to estimate an Opinion Matrix, and then summarizes the Opinion Matrix with scores for each item. This has the effect of statistically balancing out responses from users^11^. We found that the survey format was conducive to obtaining the maximal number of responses possible from the group, and was able to collect a large number of responses. We hypothesize that the relatively low number of new ideas uploaded by users was related to the robust breadth of seed ideas in the initial survey design. Prior to launching the survey, working group members had spent multiple sessions developing seed ideas, based on outreach to other members and a literature review. The two new ideas uploaded by participants, both of which included the use of automated intelligence and machine learning, received relatively low scores (Table 2, ideas marked with *). We do not have specific data regarding the number of individual respondents and time spent on the survey. However, based on our pilot survey and final results, users rated approximately 40 pairs of ideas during each session, and therefore, most respondents likely viewed and voted on most ideas presented in the survey. For future surveys, we would suggest limiting surveys to ≤ 20 ideas to maximize the number of times each idea is voted upon and allow for more research ideas to be added from respondents. Although the engineers of the wiki survey use statistical methods for mitigating respondent bias, we were unable to validate our results. If the survey is repeated on an annual basis, we expect that (1) some ideas will persist, leading to more data about validity, and (2) high priority topics will continue to evolve/change over time.

The responses collected in the final survey were similar to those we collected from the pilot; however complex CHD and conotruncal lesions received fewer votes than expected in the final survey. This likely reflects competing strengths of other imaging modalities used in pediatric cardiology (for example, echocardiography and cardiac computed tomography) in the anatomic characterization of CHD. The highest scoring gaps reflected both strengths of CMR (e.g., tissue characterization for myocardial disorders; implementation of technologic advances into clinical practice), as well as deficiencies in pediatrics (limited knowledge of normal reference values, need for early subclinical predictors due to evolution of disease during childhood). Below, we discuss these topics in more detail, as they relate to pediatric CMR.

### Myocardial disorders

We identified knowledge gaps among multiple myocardial disorders that may benefit from pediatric multicenter CMR research, including cardiac transplant rejection, myocarditis, and cardiomyopathies. This long list of disorders highlights the advantages of CMR in offering unique quantitative tissue characterization of the myocardium, which remains a limitation of other imaging modalities such as echocardiography^12^. We agree with the survey results in that survivors of orthotopic heart transplantation (OHT) could perhaps derive maximal benefit from noninvasive techniques to detect cardiac transplant rejection^13–15^. Although myocardial native T1 time and extracellular volume fraction (ECV) correlate with degree of fibrosis on endomyocardial biopsy, single center pilot studies evaluating the clinical utility of CMR in patients with OHT rejection have been limited by low statistical power due to small numbers and low rates of rejection in the study population^16–18^. Similarly, CMR appears to have great diagnostic and prognostic potential in the assessment of myocarditis and childhood cardiomyopathies. Not only does CMR provide the most reliable method for quantification of ventricular size and function, but tissue characterization techniques (such as late gadolinium enhancement imaging, native T1 and ECV) likely have important prognostic value^19–21^. Large multicenter adult studies have identified several CMR-based risk factors for sudden cardiac death in patients with hypertrophic cardiomyopathy, dilated cardiomyopathy, and arrhythmogenic right ventricular cardiomyopathy; however, these data have limited value in predicting sudden cardiac death in children, and few data exist about the role of CMR in risk prediction in children^22–27^. Children differ from adults in that the phenotypic abnormalities associated with cardiomyopathy often evolve slowly throughout the lifespan, and may be subtle or undetectable during childhood^28–30^. Tissue characteristics may provide pre-clinical markers of disease in both dilated and hypertrophic cardiomyopathy; this is particularly important in children who are transitioning from normal to abnormal myocardial architecture and mechanics.

### Implementation of new technologies & techniques into clinical practice

As a relatively new imaging modality, CMR continues to evolve with many advances in scanning and analysis techniques, as well as repurposing of older tools. However, these new technologies have not been uniformly adopted in the clinical environment, with many still being considered ‘research’ techniques. As a result, we as a specialty have been slow to achieve consensus on how or when to use technological advances for children with congenital or acquired heart disease. The following technologies that reached the highest priority are more specifically discussed below: need for sedation in infants and young children, 4-dimensional (4D) flow, and CMR in children with pacemakers and defibrillators.

#### Sedation

Traditionally, CMR protocols have required deep sedation or anesthesia for infants and young children who are unable to cooperate and breath-hold for image acquisition. Since the feed-and-sleep technique was initially described in 2012 for infants < 6 months of age, and subsequently with the introduction of newer free breathing scanning techniques that mitigate the need for breath-holding, there has been more variation in the use of sedation among different programs^31,32^. Sedation for CMR carries a number of challenges, including the need for magnetic resonance imaging (MRI)-compatible equipment, the risks of anesthesia, and the cost of extended room occupancy^33^. However, some centers continue to use sedation or anesthesia for patients with complex CHD or a need for prolonged scan protocols > 1 h^34^. Because of the large practice variation and the likely contribution of multiple patient and programmatic factors to decision-making regarding need for sedation, multicenter studies are needed to understand practice patterns, cost, efficacy, and safety of non-sedation versus sedation and general anesthesia in infants and children.

#### 4D flow

Another widely investigated tool that has not yet reached mainstream clinical care in most CMR imaging protocols is 4D flow. Particularly well-suited to the pediatric population, 4D flow allows for comprehensive flow evaluation in complex CHD, including shunt flow evaluation, vascular distensibility and flow dynamics. Perhaps even more impactful is the potential use of 4D flow as a technique for understanding valve function, intracardiac and vessel mechanics^35–39^. However, the use of 4D flow in both clinical practice and research remains limited due to time constraints and expensive software needed to handle the large volume of data. Development of a robust and shortened 4D flow sequence will improve its utility, and in the future can be an important tool beyond our current 2-dimension phase contrast flow sequences.

#### Pacemakers/defibrillators

Although CMR is a valuable imaging modality for children with complex CHD, a large proportion of patients have implantable pacemakers or defibrillators. Access to CMR for patients with implantable cardiac devices has been limited due to concerns about perceived risk and adverse clinical outcomes^40^. However, single center reports suggest that most children with pacemakers, including those with epicardial leads, can safely undergo CMR examinations^41^. The 2021 PACES expert consensus statement on the indications and management of cardiovascular implantable electronic devices in pediatric patients recommends that “MRI in all patients with conditional or non-conditional cardiovascular implantable electronic devices should be performed in the context of a defined institutional protocol”^42^. However, due to a lack of data, they are unable to provide specific recommendations or absolute contraindications to CMR in patients with epicardial or abandoned leads^43^. As pediatric CMR programs have begun to perform studies on patients with implantable cardiac devices and abandoned leads in select cases, multicenter studies are needed to better understand the safety and image quality in children.

### Normal reference values

Normative data, or normal reference values, refers to data from a reference population that establishes a baseline distribution for a measurement, and against which a measurement from an individual patient can be compared. These values can be transformed into z-scores, which are more clinically meaningful than absolute values in pediatrics, given the wide range in body size as children grow. Based on our survey results, knowledge of normal reference values for myocardial mapping, ventricular size and function, and vascular dimensions in healthy pediatric controls should be a high priority.

#### Myocardial mapping - normal reference values in children

Based on our survey, parametric mapping/tissue characterization techniques in children (including T1 and T2 mapping, and calculation of ECV), was the #1 idea scoring the highest number of votes. Although such techniques have been available since 2004^44^, their widespread implementation is limited by non-uniformity of the technique among vendors and institutions, different values based on field strength, and variability in pediatric reference values. This has presented challenges with standardization across multicenter studies, and thus affects clinical utility^45^. Clinical recommendations in adult patients with specific types of myocardial disease are derived from large multi-center studies^46^. However, there is little consensus in pediatric practice, both in terms of normal reference values and sequence optimization.

#### Normal pediatric cardiac measurements

Although several publications have reported normal parameters for pediatric anatomy and physiology in children, datasets have used different methodology and statistical techniques to obtain normal ranges and standard deviations. For example, while linear models may adequately describe the relationship between ventricular volumes and BSA in young children,, the Lamda-Mu-Sigma (LMS) method may be a more robust method to account for nonlinear relationships that occur over the life span^9,10,47–49^. Practically speaking, in order for clinical programs to implement widely accepted z-scores, we need z-score regressions that are validated across a wide range of age and BSA. Similar to statistical methods employed for echocardiographic parameters, models should account for heteroscedasticity (variation in standard deviation over time)^50^. In addition, there continues to be a paucity of data on normal reference values for free-breathing patients younger than 6 years old, with an under representation of both non-Caucasian and obese subjects^9,51,52^. The lack of reference data in younger children is in part related to their inability to lay still in a scanner without sedation. Consequently, the establishment of a large globally accepted dataset of normal parameters in children will likely require a multicenter effort gathering existing and/or prospective data from a large cross-sectional population of children.

### Multicenter research in pediatric CMR

The growing body of literature on CMR imaging biomarkers points to the need to design collaborative research studies on their predictive value to determine clinical outcomes. Due to the small numbers in single center pediatric studies, larger studies are required to study their utility as markers of disease outcome and prognosis, and to establish accurate normal reference values. Although we sought to find the highest priority gaps answerable by multicenter research, we recognize that some of these ideas may be difficult to implement. For this reason, the development of a pediatric CMR research consortium is urgently needed in order to enhance the feasibility of conducting multicenter research. We hope that this document serves as a call to industry and other stakeholders that the pediatric CMR community believes in the importance of collaboration in order to effect change.

## Limitations

By design, the wiki survey is ‘greedy’ and will allow for any number of responses from each user. Therefore, we received a varied number of votes per respondent, so that some respondents likely had more input than others. In addition, the relatively large number of seed ideas may have prevented respondents from adding their own divergent ideas. We suggest limiting the number of seed ideas in future surveys to allow for more respondent participation. Although trainees were invited to participate in the survey, no trainees were included in the original working group. Also, patients and patient families were not involved in the survey. The authors of the wiki survey acknowledge that further research is warranted to assess validity and to optimize the Opinion Matrix. Also, the anonymous nature of the survey means that a demographic or geographic representation of voters could not be obtained.

## Conclusions

The highest priorities in pediatric multicenter CMR research reflected both strengths of CMR (e.g., tissue characterization for myocardial disorders; implementation of technologic advances into clinical practice), as well as deficiencies in pediatrics (e.g., limited data on normal reference values). The wiki survey format was effective and easy to implement, and could be used for repeated surveys with modification of high priorities over time. We hope that knowledge of these high priorities will serve as a roadmap for research and funding in the field of pediatric CMR.

## Supplementary Information

Supplementary Figures.

Supplementary Tables.

## Data Availability

All data generated or analyzed during this study are included in this published article and its supplementary information files.

## Abbreviations

CMR: Cardiovascular magnetic resonance
SCMR: Society for cardiovascular magnetic resonance
CHD: Congenital heart disease
OHT: Orthotopic heart transplant
ECV: Extracellular volume fraction
4D: 4-Dimensional
MRI: Magnetic resonance imaging
