# Supplementary material for: Multicenter research priorities in pediatric CMR: results of a collaborative wiki survey
Source: Sci Rep. 2023 Jun 3;13:9022. doi: 10.1038/s41598-023-34720-9 (PMC10239463; doi:10.1038/s41598-023-34720-9)
Supplement: Supplementary file 1 — Supplementary Figures. [file 41598_2023_34720_MOESM1_ESM.docx]

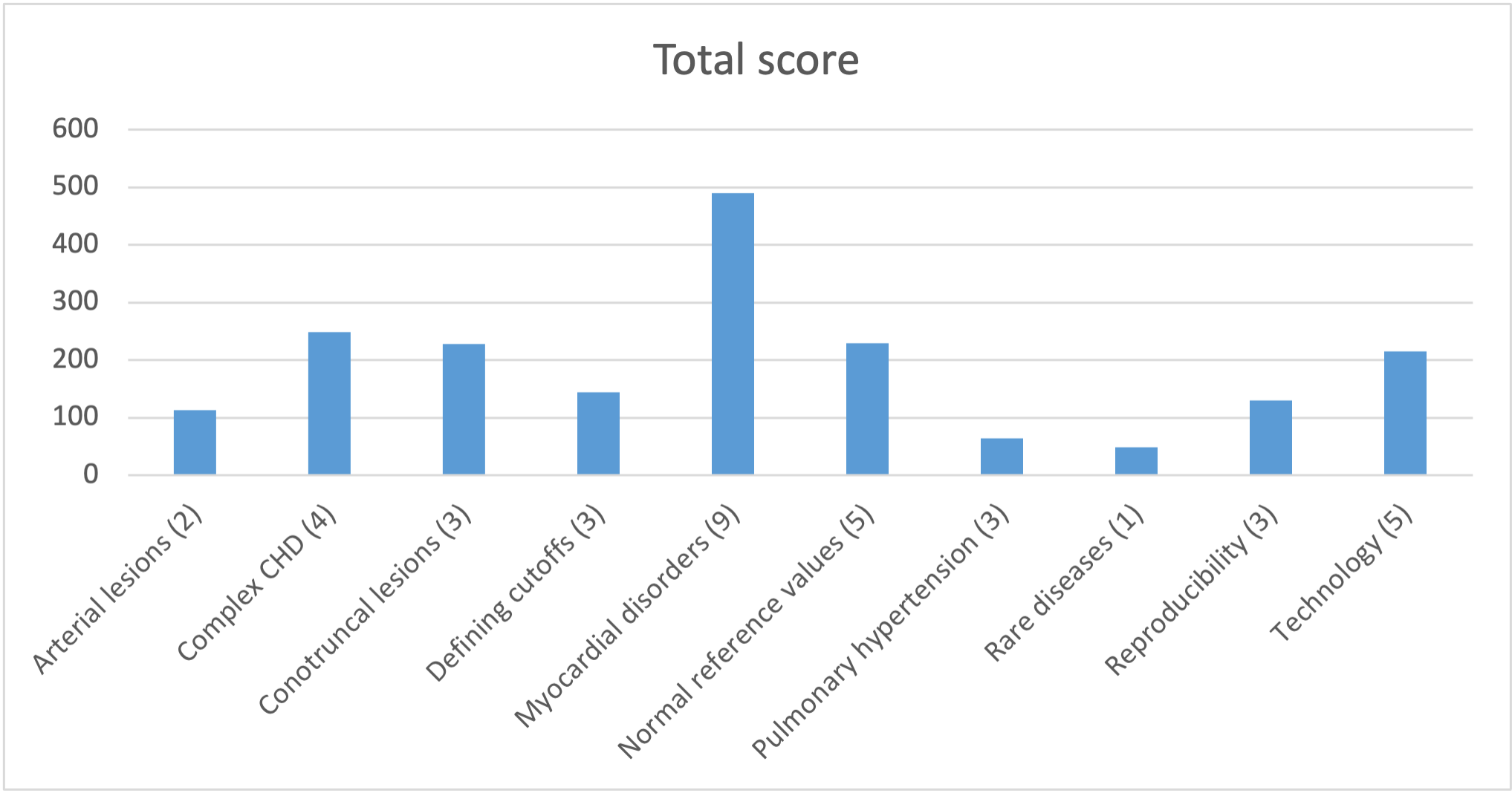


Supplemental Figure 1. Combined scores for the pilot survey based on classification. The x-axis denotes topic (#ideas), and the bar represents the combined score for all topics in that category.
