# Supplementary material for: Multicenter research priorities in pediatric CMR: results of a collaborative wiki survey
Source: Sci Rep. 2023 Jun 3;13:9022. doi: 10.1038/s41598-023-34720-9 (PMC10239463; doi:10.1038/s41598-023-34720-9)
Supplement: Supplementary file 2 — Supplementary Tables. [file 41598_2023_34720_MOESM2_ESM.docx]

Supplemental Table. Pilot survey results from the working group.

| **Multicenter research priority** | **Score** | **Classification** |
| --- | --- | --- |
| 1. TOF ACHD consensus guidelines on use of CMR: applicable in children and adolescents? | 85 | Conotruncal lesions |
| 1. TOF ACHD consensus guidelines on PVR: applicable in children and adolescents? | 79 | Conotruncal lesions |
| 1. CMR criteria for biventricular repair in borderline left ventricles | 74 | Complex CHD |
| 1. CMR predictors of outcome following the Fontan operation | 74 | Complex CHD |
| 1. Prognostic value of myocardial tissue mapping in myocarditis | 66 | Myocardial disorders |
| 1. CMR predictors of adverse outcome after the double switch operation in L-TGA | 66 | Complex CHD |
| 1. Noninvasive detection of cardiac transplant rejection in children | 65 | Myocardial disorders |
| 1. Stress CMR after arterial switch operation in D-TGA | 64 | Conotruncal lesions |
| 1. Stress CMR and LGE for assessment of anomalous aortic origin of a coronary artery (AAOCA) | 63 | Arterial lesions |
| 1. Development of a pediatric risk calculator in HCM | 59 | Myocardial disorders |
| 1. Normal ventricular mass and volumes in children | 58 | Normal reference values |
| 1. Lymphatic MR imaging: developing pathways for local implementation | 58 | Implementing new technologies & techniques into clinical practice |
| 1. Safety of CMR imaging of children with pacemakers and defibrillators | 57 | Implementing new technologies & techniques into clinical practice |
| 1. Sensitivity and specificity of CMR for detection of ARVC in children | 56 | Myocardial disorders |
| 1. Normal vascular dimensions in children | 55 | Normal reference values |
| 1. Dilated cardiomyopathy: CMR based predictors of outcome | 55 | Myocardial disorders |
| 1. Outcome after detection of LGE in children with myocarditis | 55 | Myocardial disorders |
| 1. Standardized cutoffs for aortic regurgitation and association with outcomes | 52 | Defining cutoffs for mild, moderate, severe |
| 1. Prognostic value of myocardial tissue mapping in HCM | 50 | Myocardial disorders |
| 1. CMR predictors of adverse ventricular remodeling in coarctation of the aorta | 50 | Arterial lesions |
| 1. Normal parametric imaging parameters in children by CMR and multivendor reproducibility | 49 | Normal reference values |
| 1. Parametric tissue mapping in the diagnosis of cardiac tumors and masses | 48 | Rare diseases |
| 1. Consensus on how to measure dilated aortas | 47 | Defining cutoffs for mild, moderate, severe |
| 1. Prognostic value of CMR in pediatric cancer survivors | 47 | Myocardial disorders |
| 1. Clinical utility of 4D flow | 46 | Implementing new technologies & techniques into clinical practice |
| 1. Stress CMR: institutional variation in stress, imaging protocols, interpretation | 45 | Reproducibility |
| 1. Standardized cutoffs for AVV regurgitation and association with outcomes | 45 | Defining cutoffs for mild, moderate, severe |
| 1. Reproducibility of LGE quantification and association with outcomes | 45 | Reproducibility |
| 1. Reproducibility of ventricular measurements (function, mass) across institutions | 40 | Reproducibility |
| 1. Need for sedation in infants & young children | 38 | Implementing new technologies & techniques into clinical practice |
| 1. Prognostic value of CMR in Duchenne muscular dystrophy-associated cardiomyopathy | 37 | Myocardial disorders |
| 1. Normal atrial size in children | 34 | Normal reference values |
| 1. Does veno-venous collateral burden predict desaturation and exercise capacity in Fontan patients? | 34 | Complex CHD |
| 1. Nomogram for dilated aorta in Turner syndrome | 33 | Normal reference values |
| 1. Is exercise CMR prognostic in children with pulmonary arterial hypertension? | 29 | Pulmonary hypertension |
| 1. CMR-guided catheterization for assessment of pulmonary arterial hypertension | 23 | Pulmonary hypertension |
| 1. Clinical utility of fetal CMR | 19 | Implementing new technologies & techniques into clinical practice |
| 1. CMR before and after the Potts shunt for pulmonary arterial hypertension | 12 | Pulmonary hypertension |
